# Myosteatosis as a metabolic risk in hidradenitis suppurativa: a CT-based study

**DOI:** 10.3389/fmed.2025.1719183

**Published:** 2026-01-06

**Authors:** Didem Didar Balci, Aygün Talibova, Caner Öztürk, Yağız Üstündağ, Raif Can Yarol, Melis Gönülal, Emine Argüz, Melih Özcan, Burçin Kibar Öztürk, Gül Çolakoğlu, Veli Süha Öztürk, Ali Balci

**Affiliations:** 1 Department of Dermatology, Faculty of Medicine, Dokuz Eylül University, İzmir, Türkiye; 2 Department of Radiology, Faculty of Medicine, Dokuz Eylül University, İzmir, Türkiye; 3 Department of Dermatology, Tepecik Training and Research Hospital, İzmir, Türkiye; 4 Department of Radiology, Tepecik Training and Research Hospital, İzmir, Türkiye; 5 Department of Radiology, Faculty of Medicine, Ondokuz Mayıs University, Samsun, Türkiye

**Keywords:** hidradenitis suppurativa, myosteatosis, sarcopenia, metabolic comorbidities, intermuscular adipose tissue, computed tomography

## Abstract

**Background:**

Hidradenitis suppurativa (HS) is a chronic inflammatory skin disorder linked to obesity, metabolic syndrome, and systemic inflammation. Myosteatosis is associated with adverse metabolic outcomes, but its relationship with HS has not been explored. The present study aimed to compare muscle and fat composition, focusing on myosteatosis and sarcopenia, between HS patients and controls, and to assess associations with clinical and metabolic parameters.

**Methods:**

This retrospective study included 61 HS patients and 59 age- and sex-matched controls with non-contrast thoracoabdominal computed tomography (CT) scans. Muscle and fat compartments at the 12th thoracic vertebra were quantified. Sarcopenia and myosteatosis were defined using established CT-based criteria. Clinical and laboratory data, including fasting glucose, lipid profile, and comorbidities, were collected.

**Results:**

According to logistic regression analyses, myosteatosis showed significant independent associations with HS and with a history of biologic therapy (HS: OR = 3.88, *p* = 0.004; biologic therapy: OR = 0.085, *p* = 0.014). HS patients exhibited significantly higher intermuscular adipose tissue (IMAT) (1574.8 ± 1243.8 cm^2^ vs. 1112.7 ± 822.5 cm^2^, *p* = 0.029), low attenuation muscle area (LAMA) (5013.1 ± 1999.8 cm^2^ vs. 2996.4 ± 1220.7 cm^2^, *p* < 0.001), and LAMA/BMI indices (low attenuation muscle area normalized to BMI), alongside lower normal attenuation muscle area (NAMA)/BMI indices (*p* < 0.001), indicating increased myosteatosis and reduced muscle quality. Sarcopenia prevalence was similar. Hypertensive patients had lower NAMA/TAMA (total abdominal muscle area) and higher LAMA/BMI indices. Disease severity negatively correlated with HDL cholesterol (*p* < 0.001). BMI, TAMA, subcutaneous, and visceral adipose tissue were similar between groups.

**Conclusion:**

This study demonstrates that HS is independently associated with myosteatosis, but not with sarcopenia. A history of long-term biologic therapy was also strongly and independently linked to myosteatosis, suggesting a previously underrecognized interaction between systemic inflammation, treatment exposure, and muscle quality. Muscle fat infiltration may represent an underrecognized metabolic risk factor in HS, highlighting the need for further prospective studies.

## Introduction

Hidradenitis suppurativa (HS) is a chronic inflammatory skin disorder characterized by recurrent, painful nodules, abscesses, fistulas, and scarring, predominantly affecting intertriginous areas such as the axillae, inguinal region, gluteal region, and inframammary folds (1). The prevalence of HS ranges from 0.03 to 4.1%, a variability largely explained by differences in diagnostic criteria, population demographics, and underrecognition of the disease. HS is more common in women, usually appears after puberty, and is strongly associated with modifiable risk factors such as smoking and obesity, as well as genetic predisposition (1–4).

Beyond its cutaneous manifestations, HS is increasingly recognized as a systemic inflammatory condition with metabolic comorbidities such as type 2 diabetes, obesity, dyslipidemia and hypertension (5, 6). This chronic inflammatory burden, together with lifestyle and metabolic factors, raises the possibility that HS may influence muscle composition, including myosteatosis and sarcopenia, providing the rationale for investigating these parameters in affected patients. Obesity represents a key driver of chronic low-grade systemic inflammation. Dysregulation of adipokine secretion from adipose tissue—characterized by elevated levels of pro-inflammatory mediators such as resistin, visfatin, and leptin, alongside decreased levels of the anti-inflammatory adiponectin—appears to contribute to the pathogenesis of HS (1). Supporting this concept, a recent study employing bioelectrical impedance analysis demonstrated increased total body fat and visceral adiposity, coupled with reduced predicted muscle mass, in HS patients compared with controls. Notably, however, this study did not address muscle quality or myosteatosis (7).

Myosteatosis refers to the pathological infiltration of fat within skeletal muscle, encompassing intramuscular (between fibers), intermuscular (between fascicles), and intramyocellular (within myocytes) compartments (8). In contrast, sarcopenia is defined as the progressive and degenerative decline in skeletal muscle mass, quality, and strength, primarily associated with aging and physical inactivity. Chronic low-grade inflammation and “inflammaging” are recognized as key contributors to the deterioration of muscle mass and function (9). Although myosteatosis and sarcopenia frequently coexist, they represent distinct clinical entities. Both conditions can be reliably assessed using cross-sectional imaging techniques, particularly computed tomography (CT) and magnetic resonance imaging (MRI) (10, 11). Among these, the quantification of muscle and fat distribution at the level of the twelfth thoracic vertebra has emerged as a standardized and widely adopted approach (11). Importantly, myosteatosis has been consistently linked not only to chronic low-grade inflammation but also to adverse metabolic outcomes, including metabolic syndrome, insulin resistance, and type 2 diabetes mellitus (10).

To the best of our knowledge, there are no studies in the literature evaluating the potential relationship between HS and myosteatosis or sarcopenia. We hypothesized that HS patients exhibit increased myosteatosis and reduced muscle quality compared with controls, reflecting systemic inflammation and metabolic dysregulation.

## Methods

### Study population

The study retrospectively included patients aged 18 years or older who were diagnosed with HS at the Dokuz Eylül University Medical Faculty, Dermatology Clinic between January 1, 2014, and April 1, 2025. From a total of 555 HS patients, 74 subjects with at least one non-contrast thoracic and/or abdominal CT scan available in the PACS system were initially identified. Contrast-enhanced CT scans were excluded from the study due to their potential impact on tissue density measurements. Exclusion criteria included a history of malignancy, systemic steroid use, chronic kidney disease, clinically detectable edema, prior abdominal or spinal surgery, anatomical distortion, or CT scans of insufficient image quality. Patients with chronic kidney disease were excluded to avoid potential alterations in tissue characteristics and imaging quality associated with renal impairment. Those with prior abdominal or spinal surgery were excluded to prevent anatomical distortions that could compromise accurate muscle and fat measurements. After applying these criteria—excluding patients with malignancy (*n* = 6), systemic steroid use (*n* = 3), chronic kidney disease (*n* = 2), or prior abdominal or spinal surgery (*n* = 2)—the final study cohort comprised 61 patients. HS diagnosis was established by experienced dermatologists based on widely accepted clinical criteria, including typical lesion morphology, location in intertriginous areas, chronicity, and recurrence. This approach aligns with recognized diagnostic standards in clinical practice. Disease severity was assessed using the Hurley staging system. Demographic characteristics, laboratory results, and clinical findings recorded at the time of CT imaging or the closest available time point were obtained from the hospital information system. The most recent CT scan was incorporated into the analysis, and disease duration was determined based on its timing. CT scans were performed for various clinical indications unrelated to HS, such as pneumonia, abdominal pain, abdominal infection, dyspnea, or malignancy screening. Therefore, imaging was not standardized relative to the time of HS diagnosis. Controls were randomly selected from a pool of patients who underwent CT scans for suspected urinary tract stones but exhibited no pathological findings and were matched to the study patients with respect to age and gender. Information regarding BMI, smoking status, and metabolic comorbidities was not obtained for the control group.

The study protocol was approved by the Institutional Review Board of our university (GOA, 2025/24-21) and all procedures were conducted in accordance with the principles of the Declaration of Helsinki.

### CT image analysis

CT scans were acquired using two multidetector scanners (Toshiba Aquilion PRIME and Philips Ingenuity CT). All thoracic and/or abdominal CT examinations were acquired using standardized institutional imaging protocols, including same slice thickness, reconstruction kernels, and tube voltage settings. Both scanners undergo routine manufacturer-recommended calibration as part of the radiology department quality assurance program, and therefore no additional post-processing harmonization procedures were required. Muscle and fat measurements were performed in a blinded manner using 3D-Slicer (v5.6.1) by a single trained radiologist to minimize bias. On the transverse slice at the 12th thoracic vertebra (Th12), the psoas, paraspinal (erector spinae, quadratus lumborum), and abdominal muscles (transversus abdominis, external and internal obliques, rectus abdominis) were manually segmented with Hounsfield Unit (HU) thresholds set between −29 and 150. Muscle areas were calculated in cm^2^ (12).

**Figure 1:**
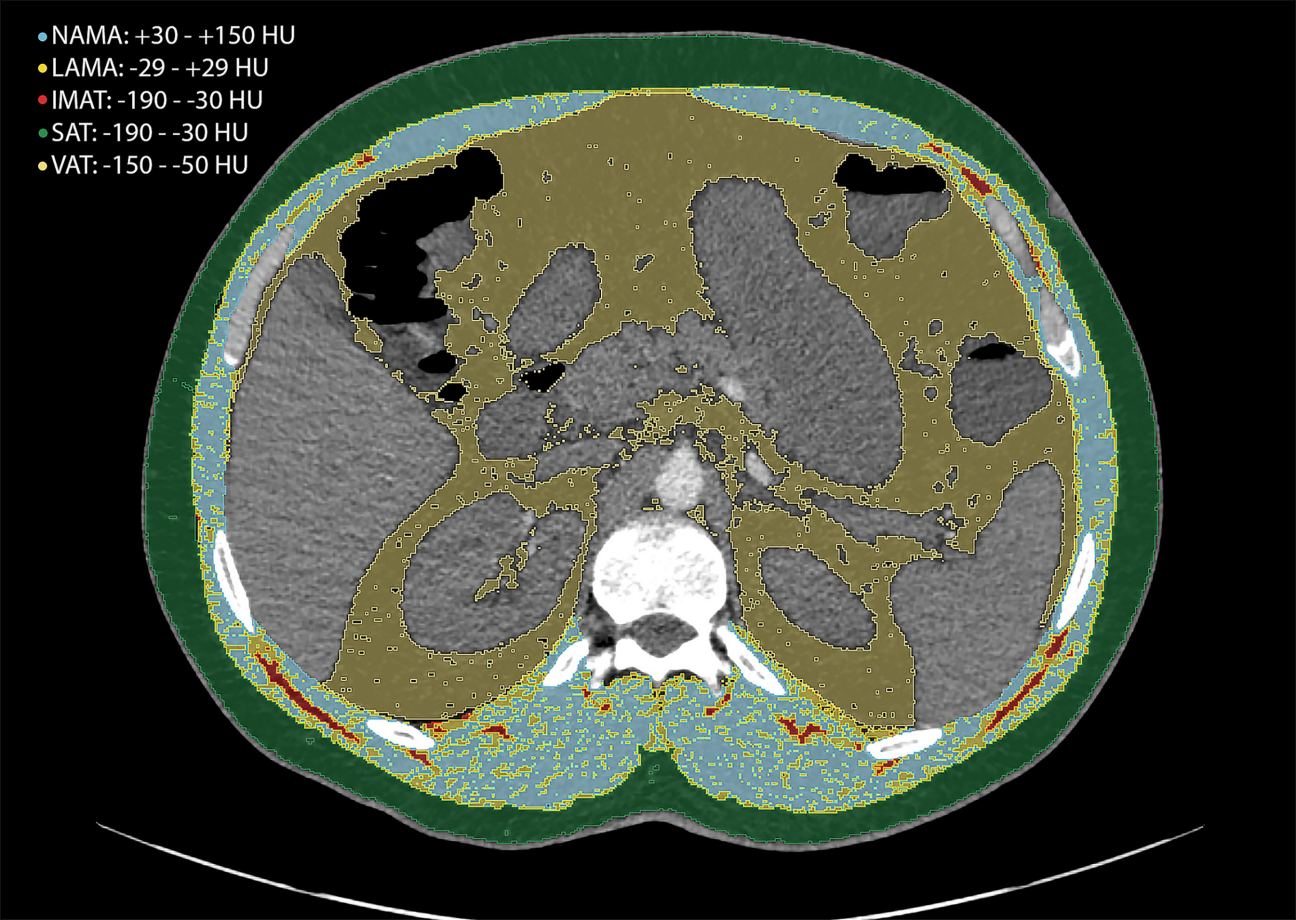
Computed tomography (CT) images at the level of the 12th thoracic vertebra were used for the quantification of intermuscular adipose tissue (IMAT), normal attenuation muscle area (NAMA), low attenuation muscle area (LAMA), subcutaneous adipose tissue (SAT), and visceral adipose tissue (VAT), expressed in Hounsfield units (HU).

Skeletal muscle mass was assessed according to established criteria. The skeletal muscle index (SMI, cm^2^/m^2^) was calculated as total skeletal muscle area (SMA, cm^2^) divided by height squared (m^2^), with sarcopenia defined as SMI < 25.75 cm^2^/m^2^ in men and < 20.16 cm^2^/m^2^ in women. Intermuscular adipose tissue percentage (IMAT%) was calculated as (IMAT / SMA) × 100, and myosteatosis was defined as IMAT% > 7.51 in men and > 6.83 in women. IMAT (−190 to −30 HU) represents the apparent fat tissue between muscle groups and muscle fibers (12). The normal-attenuation muscle area (NAMA) and low-attenuation muscle area (LAMA), were calculated using standardized Hounsfield unit thresholds: NAMA (+30 to +150 HU) representing nonfatty muscle with little intramuscular fat, LAMA (−29 to +29 HU) (10–12) representing fatty muscles with intramuscular lipid (Figure 1).

### Laboratory parameters

Clinical data, including age, sex, height, weight, smoking status, alcohol consumption, hypertension, and diabetes mellitus, were obtained from the hospital information system. Laboratory measurements in HS patients included fasting blood glucose, total cholesterol (TC), triglycerides (TG), high-density lipoprotein cholesterol (HDL-C), low-density lipoprotein cholesterol (LDL-C), and C-reactive protein (CRP).

### Statistical analysis

Continuous data are expressed as mean ± SD and categorical data as percentages. Normality was assessed with the Kolmogorov–Smirnov test. Continuous variables were compared between the two groups using either the independent *t*-test or the Mann–Whitney U test, as appropriate. Categorical variables were compared between the two groups using the chi-square test. The associations among variables were analyzed using the Spearman correlation test. Logistic regression analyses were performed to evaluate the independent associations between myosteatosis and laboratory or clinical features, adjusting for potential confounders. A two-sided *p*-value <0.05 was considered statistically significant. Analyses were conducted with SPSS v24 (SPSS Inc., Chicago, IL, United States).

Because all eligible cases within the predefined study period were included, *a priori* sample size or power calculations were not undertaken. Missing data were handled by excluding cases with incomplete information from the relevant analyses; no imputation was performed.

## Results

Table 1 presents the main demographic characteristics and CT parameters of the study participants. Mean values of SMD, NAMA, LAMA, IMAT, as well as the NAMA/TAMA, NAMA/BMI, and LAMA/BMI indices were significantly different from those of the control group. The prevalence of sarcopenia was similar between the groups; however, the prevalence of myosteatosis was significantly higher in patients with HS (Table 1).

**Table 1:** Demographic features and computed tomography measurements of the subjects.

Variable	HS patients (*n* = 61)	Controls (*n* = 59)	*p***-**values
Age (year)	48.0 ± 14.1	48.1 ± 13.8	0.973*
Male/female (number)	49/12	48/11	0.931‡
BMI (kg/m^2^)	28.1 ± 4.9	26.9 ± 2.9	0.130**
Myosteatosis (number)	45 (73.8%)	30 (50.8%)	0.014‡
Sarcopenia (number)	1 (1.6%)	1 (1.7%)	0.981‡
SMI (cm^2^/m^2^)	41.3 ± 8.5	44.1 ± 11.1	0.148**
TAMA (cm^2^)	13956.4 ± 3526.0	14428.0 ± 4175.7	0.505**
SMA (cm^2^)	12410.4 ± 2903.5	13089.7 ± 3527.0	0.279**
SAT (cm^2^)	11884.6 ± 6317.4	14145.9 ± 6836.7	0.05*
VAT (cm^2^)	12034.7 ± 8438.0	13593.1 ± 8472.9	0.271*
SMD (HU)	36.4 ± 8.8	37.6 ± 9.9	0.451**
NAMA (cm^2^)	7539.1 ± 2505.3	10261.9 ± 3659.1	<0.001**
LAMA (cm^2^)	5013.1 ± 1999.8	2996.4 ± 1220.7	<0.001*
IMAT (cm^2^)	1574.8 ± 1243.8	1112.7 ± 822.5	0.029*
SMA/BMI	447.5 ± 98.8	484.8 ± 132.1	0.081**
NAMA/BMI	272.9 ± 89.6	381.1 ± 129.9	<0.001**
LAMA/BMI	179.8 ± 67.6	110.3 ± 40.3	<0.001*
NAMA/TAMA (%)	54.0 ± 14.4	70.1 ± 10.9	<0.001**

BMI, body mass index; SMI, skeletal muscle index; TAMA, total abdominal muscle area; SMA, skeletal muscle area; SAT, subcutaneous adipose tissue; SMD, skeletal muscle density; VAT, visceral adipose tissue; NAMA, normal attenuation muscle area; LAMA, low attenuation muscle area; IMAT, intermuscular adipose tissue. *Mann–Whitney U test, ** Independent *t* test, ‡ Chi-square test.

The main clinical and laboratory characteristics of patients with HS are presented in Table 2.

**Table 2:** Clinical and laboratory features of the HS patients.

Disease duration (year)	12.8 ± 9.7
Hurley grade (number)	
Grade 1	3 (4.9%)
Grade 2	28 (45.9%)
Grade 3	30 (49.2%)
Alcohol drinker (number)	
None	32 (52.5%)
Once a week	27 (44.2%)
Regular	2 (3.3%)
FBS (mg/dL)	99.3 ± 15.6
Diabetes mellitus (number)	16 (26.2%)
Hypertension (number)	21 (34.4%)
LDL cholesterol (mg/dL)	117.4 ± 33.5
HDL cholesterol (mg/dL)	42.5 ± 10.4
Triglyceride (mg/dL)	156.6 ± 149.0
Total cholesterol (mg/dL)	190.8 ± 47.3
CRP (mg/dL)	32.1 ± 44.7

FBS, fast blood sugar; LDL, low-density lipoprotein; HDL, high-density lipoprotein; CRP, C-reactive protein.

Tables 3, 4 present the variables that show an independent association with myosteatosis. In the logistic regression analysis including all patients, myosteatosis demonstrated an independent association with the presence of HS, whereas in the subgroup analysis restricted to HS patients, myosteatosis was independently associated with a history of biologic use. No independent association was identified between myosteatosis and diabetes, smoking, hypertension, laboratory parameters or disease severity (Hurley grade) (*p* > 0.05).

**Table 3:** Logistic regression analysis of factors associated with myosteatosis in the entire group of the 120 study subjects.

Variable	OR	95% CI	*p* value
Sex (male)	6.218	1.620–23.870	0.008
BMI	1.261	1.097–1.450	0.001
HS	3.880	1.535–9.811	0.004
Age	1.071	1.034–1.109	<0.001

OR, odds ratio; CI, confidence interval; BMI, body mass index; HS, hidradenitis suppurativa.

**Table 4:** Logistic regression analysis of factors associated with myosteatosis in the 61 HS patients.

Variable	OR	95% CI	*p* value
Sex (male)	0.09	0.000–0.446	0.018
BMI	1.625	1.197–2.208	0.002
History of biologic therapy	0.085	0.012–0.602	0.014
Age	1.110	1.028–1.198	0.07

OR, odds ratio; CI, confidence interval; BMI, body mass index.

In a subgroup analysis of HS patients, disease severity (Hurley grade) was negatively correlated with HDL cholesterol levels (r = −0.560, *p* < 0.001) and IMAT (r = −0.268, *p* = 0.037). Fasting blood glucose levels were positively correlated with VAT (r = 0.279, *p* = 0.032) and negatively correlated with SMD (r = −0.285, *p* = 0.029).

Among diabetic HS patients, NAMA (*p* = 0.024), the NAMA/TAMA ratio (*p* = 0.029), the SMI/BMI ratio (*p* = 0.037), and the NAMA/BMI ratio (*p* = 0.005) were significantly lower compared to non-diabetic HS patients. In the comparison between HS patients with and without hypertension, SMD (*p* = 0.07) and the NAMA/TAMA index (*p* = 0.014) were significantly lower in hypertensive patients, whereas the LAMA/BMI index (*p* = 0.023) and LAMA (*p* = 0.015) were significantly higher.

At the time of the study, 7 (11.5%) patients were using adalimumab, 1 (1.6%) certalizumab, 6 (9.8%) infliximab, 10 (16.4%) oral antibiotherapy, 8 (13.1%) topical antibiotherapy and 1 (1.6%) acitretin. The remaining 28 (45.9%) were not taking any therapy. No significant association was found between current therapies and alcohol consumption status, smoking and CT parameters or the presence of myosteatosis and sarcopenia (*p* > 0.05).

HS patients who had received at least one biologic agent for a minimum of twelve months in their treatment history were classified as biologic-positive according to the CT scan time. A total of 23 patients had a history of biologic therapy for at least 12 months. Among them, 13 had been treated with adalimumab, 3 with infliximab, 4 with both adalimumab and infliximab, 1 with adalimumab, infliximab, and secukinumab, 1 with adalimumab, infliximab, secukinumab, and anakinra, and 1 patient had received secukinumab, infliximab, and anakinra. Logistic regression analysis demonstrated that biologic positivity was independently associated with myosteatosis (Table 4).

## Discussion

In our study, we identified an independent association between HS and myosteatosis, representing one of the first investigations of this relationship. Notably, logistic regression analysis revealed that biologic therapy use was independently associated with significantly lower odds of myosteatosis compared with patients not receiving biologic agents (OR = 0.085, *p* = 0.014), suggesting a strong protective effect in HS patients. This finding indicates that biologic exposure may mitigate intramuscular fat accumulation in HS patients, potentially through reduction of systemic inflammation. Although data in HS are limited, however, data from other chronic inflammatory conditions provide important context. Systemic inflammation—driven by cytokines such as TNF-*α*, IL-1β, and IL-6—is known to promote muscle catabolism and intramuscular lipid accumulation, thereby contributing to both sarcopenia and myosteatosis. Studies in rheumatoid arthritis have shown that biologic therapies targeting these cytokines can significantly alter body composition: IL-6 inhibition with tocilizumab has been associated with increases in lean mass and favorable metabolic shifts, whereas anti-TNF therapy has demonstrated improvements in muscle mass and density in some cohorts (13). A pilot study of TNF-inhibitor therapy showed that after 6 months, lean mass improved significantly in RA patients (14).

TNF-*α* and IL-17 are central cytokines in the pathogenesis of HS. HS patients exhibit significantly elevated TNF-α levels compared to healthy controls, which correlate positively with disease severity (15, 16). Myosteatosis may contribute to a metabolically pro-inflammatory state by releasing adipokines such as leptin, adiponectin, IL-6, and active TNF-α. Large cohort studies have demonstrated that myosteatosis is positively associated with CRP, IL-6, and resistin levels, while showing a negative association with adiponectin (10). Furthermore, in chronic conditions such as chronic obstructive pulmonary disease (COPD), muscle fat infiltration has been strongly linked to systemic inflammation, highlighting a potential mechanistic connection between adipose tissue infiltration and chronic inflammatory processes (17). A meta-analysis including 17 studies with 11,249 participants demonstrated that CRP levels were significantly elevated in sarcopenic individuals, whereas differences in IL-6 and TNF-*α* were not statistically significant (18). In our study, no significant difference in sarcopenia was observed between HS patients and controls, a finding closely mirroring results from a recent psoriasis study (19). Although HS and psoriasis differ both clinically and pathogenetically, they share common features, including the initiation of inflammatory pathways, a determinative genetic predisposition, and immune system hyperactivation. The IL-23/Th17 axis and TNF-α are key shared mediators in both conditions (20).

Beyond muscle mass, we also assessed NAMA, TAMA, and the NAMA/TAMA indices. HS patients exhibited a significantly lower NAMA/TAMA index compared to controls, indicating reduced muscle quality. Supporting this, a large cross-sectional study reported that metabolically healthy individuals had higher NAMA and NAMA/TAMA index values than metabolically unhealthy counterparts. Notably, in non-obese participants, the decreased NAMA/TAMA index was independently associated with metabolically unhealthy phenotypes. These findings underscore that maintaining metabolic health relies not only on muscle quantity but also on muscle quality (21).

In a subgroup analysis of HS patients, logistic regression analysis revealed no independent association between diabetes mellitus and myosteatosis. However, descriptive analyses indicated that nearly all diabetic HS patients, except for one, exhibited myosteatosis. Diabetic HS patients demonstrated significantly lower NAMA, NAMA/TAMA, SMI/BMI and NAMA/BMI indices—parameters that robustly quantify myosteatosis—and higher LAMA indices compared to non-diabetic HS patients. NAMA, NAMA/TAMA, and NAMA/BMI indices tended to be lower, and LAMA indices tended to be higher among diabetic HS patients. Fasting blood glucose levels showed trends consistent with increased intramuscular fat, but correlations were not significant after adjusting for potential confounders. These findings highlight the potential interplay between insulin resistance and myosteatosis in HS, suggesting that ectopic fat accumulation may contribute to altered glucose metabolism, although causality cannot be inferred. Current evidence further indicates that aging, poor nutrition, oxidative stress, insulin resistance, and inflammation may synergistically drive myosteatosis development (10, 22).

The relationship between myosteatosis and hypertension is well recognized. In a cohort of 19,766 individuals, the risk of hypertension was reported to be 2.3-fold higher in men and 2.6-fold higher in women within the lowest NAMA/BMI index quartile (23). Although no independent association between myosteatosis and hypertension was observed in our HS group, Patients with hypertension exhibited significantly lower NAMA/TAMA index and SMD values, indicative of healthy muscle mass, while LAMA and LAMA/BMI indices, markers of fat-infiltrated muscle associated with myosteatosis, were markedly elevated. These findings further underscore a potential link between hypertension and myosteatosis-related muscle alterations in HS patients, but causality cannot be inferred.

HS disease severity was negatively correlated with HDL cholesterol levels (r = −0.560, *p* < 0.001). Beyond its role in lipid transport, HDL may modulate inflammation, and its associated enzyme PON1, which possesses anti-inflammatory properties, is reduced in HS patients compared to controls (24). These findings suggest that impaired HDL function could contribute to the heightened inflammatory state in HS, highlighting the potential relevance of HDL-related mechanisms in disease pathophysiology. Our study has several key limitations. First, the relatively small sample size and single-center design may limit the generalizability of our findings. Second, the retrospective and cross-sectional nature of the study precludes evaluation of temporal relationships and causal inference. Control subjects were selected from individuals undergoing imaging for suspected for urinary system stones rather than healthy individuals which may introduce selection bias and influence metabolic characteristics independent of HS. In our hospital, healthy individuals undergoing routine check-ups do not undergo CT as part of the evaluation protocol. Performing CT scans, which involve radiation exposure, on healthy individuals was considered ethically inappropriate. Additionally, laboratory data, detailed information regarding BMI, smoking status, or subclinical inflammatory/metabolic conditions was not consistently available for the control group and important confounders, such as physical activity habits and dietary factors, could not be analyzed. Although T12-level assessments are widely used and validated for estimating muscle composition, they may not perfectly reflect whole-body muscle quality or adipose distribution. Finally, although HS is known to be more common in women, the majority of patients in our study were male.

## Conclusion

In conclusion, our study indicated that HS is independently associated with myosteatosis, but not with sarcopenia. Furthermore, HS patients with at least 1 year of biologic agent exposure exhibited a strong independent association with myosteatosis, highlighting a previously underexplored link between systemic inflammation, biologic therapy, and skeletal muscle alterations. Several myosteatosis parameters were also significantly associated with both diabetes mellitus and hypertension in HS patients. Taken together, these findings underscore the clinical importance of monitoring muscle composition in HS patients and provide a strong rationale for future prospective, longitudinal studies with larger sample sizes to elucidate the causal pathways linking systemic inflammation, metabolic disorders, biologic treatment, and myosteatosis.

## Data Availability

The raw data supporting the conclusions of this article will be made available by the authors, without undue reservation.
